# Advances in detection of infectious agents by aptamer-based technologies

**DOI:** 10.1080/22221751.2020.1792352

**Published:** 2020-07-20

**Authors:** Hui-Yan Li, Wan-Nan Jia, Xin-Yi Li, Li Zhang, Chang Liu, Jian Wu

**Affiliations:** a Department of Medical Microbiology, MOE/NHC/CAMS Key Laboratory of Medical Molecular Virology, School of Basic Medical Sciences, Fudan University, Shanghai, People’s Republic of China; b Department of Gastroenterology & Hepatology, Zhongshan Hospital of Fudan University, Shanghai, People’s Republic of China; c Shanghai Institute of Liver Diseases, Fudan University Shanghai Medical College, Shanghai, People’s Republic of China

**Keywords:** Aptamer, HBV, HCV, HIV, tuberculosis, leishmania, amoeba

## Abstract

Infectious diseases still remain one of the biggest challenges for human health. Accurate and early detection of infectious pathogens are crucial for transmission control, clinical diagnosis, and therapy. For a traditional reason, most immunological and microbiological laboratories are equipped with instruments designated for antibody-based assays in detection of infectious pathogens or clinical diagnosis. Emerging aptamer-based technologies have pushed a shift from antibody-based to aptamer-based assays due to equal specificity, even better sensitivity, lower manufacturing cost and more flexibility in amending for chemiluminescent, electrochemical or fluorescent detection in a multifaceted and high throughput fashion in comparison of aptamer-based to antibody-based assays. The nature of aptamer-based technologies is particularly suitable for point-of-care testing in remote areas at warm or hot atmosphere, and mass screening for potential infection in pandemic of emerging infectious agents, such as SARS-CoV or SARS-CoV-2 in an epicentre or other regions. This review intends to summarize currently available aptamer-based technologies in detection of bacterial, viral, and protozoan pathogens for research and clinical application. It is anticipated that potential technologies will be further optimized and validated for clinical translation in meeting increasing demands for prompt, precise, and reliable detection of specific pathogens in various atmospheric conditions.

## Introduction

In a highly mobile, interdependent but interrelated world, infectious diseases spread so rapidly that they pose a serious global threat to public health [1]. Rapid, convenient, and high-throughput testing is a key to control the transmission of emerging infectious agents. Antibodies have been used in microbial immunoassay for more than 50 years [2]. Their inherent ability to bind microbial preparations and their products makes them ideal to capture molecules for immunoassay, such as ELISA, immunochromatographic bands, column chromatography, and immunosensor development [3,4]. However, there is an increasing need for affordable, rapid, sensitive, and high throughput methods in identifying new or re-emerging infectious agents or distinguishing resistant subclones.

Nucleic acid aptamer is small single-stranded DNA (ssDNA) or RNA, identified and selected from a synthetic oligonucleotide library with a length of 20–80 bases via systematic evolution of ligands by exponential enrichment (SELEX) or other modified SELEX strategies [5]. Aptamers have been considered to be “artificial antibodies,” and considerable attention has been given to nucleic acid aptamers because of their low production cost, easy chemical modification, high chemical stability and binding affinity, repeatability, and reusability. It may be folded into secondary and tertiary spatial structure with a combination of loops, stems, hairpins, pseudoknots, bumps, or G-quadruplexes. This feature gives them high specificity and affinity to a variety of targets, ranging from ions to molecules of various properties, sizes, and complexities throughout a cell, including small molecules (such as amino acids, nucleotides, antibiotics), macromolecules (peptides, proteins, nucleic acids), or whole viruses, bacteria, and cells, etc. The advantages of aptamers in biological use are concisely highlighted in Table 1. Aptamers are equally applicable in many assays where antibodies are used, and they are even more flexible and amendable when real-time detection is needed or for on-site detection when a refrigerator is unavailable. Those advantages make it possible to be a detecting probe for infectious agents.

**Table 1.** Bullet points for aptamer advantages.

High affinity with dissociation constant (K_D_) in a nanomolar-to-picomolar range High binding specificity for a wide range of targets Synthesis *in vitro* with high purity and a low cost Flexible modification by a variety of chemical markers Small molecular weight, good stability and renewability Easily to be developed in multifaceted and high throughput assay format

Because of the unique advantages in capable of diagnosis, aptamers have been used in the design of biosensors applicable in infectious diseases over the past 20 years. The technology of detecting infectious diseases based on aptamers has achieved tremendous progress and is becoming a promising detection strategy in recent 10 years. Since its first application in 1990 [6], SELEX has undergone many modifications. The novel modification makes the obtained aptamer more specific, the selection process more efficient, cost-effective, and greatly reduces time consumption [7]. Specific aptamers for infectious microorganisms in the event of new epidemic outbreaks can be quickly obtained through efficient SELEX technology, such as the recent specific aptamers of SARS-CoV-2 have been obtained rapidly [8]. Once the specific aptamers of pathogenic microorganisms or their toxic molecules are identified, a variety of detection schemes can be designed based on the aptamers, including chemiluminescent, electrochemical or fluorescent detection according to the different detection signals [9–12]. In order to improve the sensitivity of aptamer-based detection, several research teams tried to develop signal amplification strategies. Three methods, catalytic molecule, cyclic enzymatic, and rolling-circle amplification (RCA) are used to amplify signals for better detection and a breakthrough has been made in the sensitivity of aptamer-based infectious agents detection [13–15]. Through the acquisition of specific aptamers of pathogenic microorganisms and the establishment of sensitive and stable aptamer-based detection methods, it is possible to diagnose infectious agents conveniently, quickly, and effectively in any environment.

**Figure 1.**
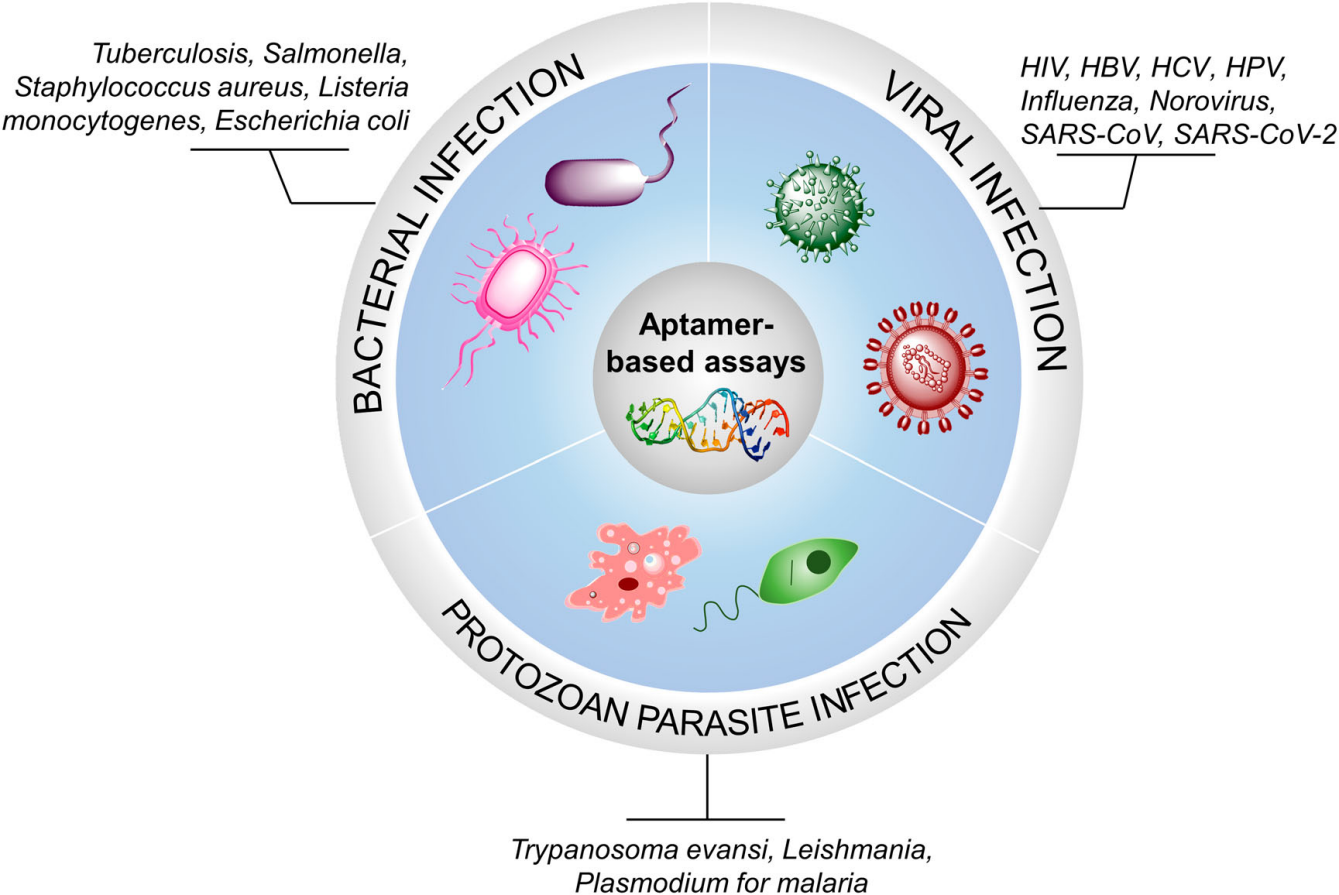
Pathogens potentially detectable by aptamer-based assays. Pathogens which are possibly detected by aptamer-based assays are categorized into three major microorganisms, bacteria, viruses and protozoan parasites.

This review intends to provide an update in the use of aptamer-based technologies in detection of infectious agents, and to demonstrate the flexibility and applicability of these technologies in precise detection of many categories of microbial agents, such as pathogenic bacteria, viruses, parasites or their products in a solid or liquid phase (Figure 1). In-depth technical overviews regarding emerging aptamer-based biosensors and cutting-edging technologies for the detection of proteins from various pathogens are available [16,17]. Representative aptamer-based methods for pathogenic targets from recent literature are briefly discussed in the text and the remaining is mentioned in Tables 2–3.

**Table 2.** Aptamers used in detection of bacteria and their products.

Bacterial types	Aptamer	Target	Method
Tuberculosis [9]	H63SL2-M6	HspX	Aptamer linked immobilized sorbent assay and electrochemical sensor
Tuberculosis [18]	Thiolated MPT64 aptamer	MPT64	Combination of aptamers and electrochemical impedance spectroscopy
Tuberculosis [19]	MAA I and MAA II	MPT64	A sandwich-type electrochemical aptasensor
Salmonella [10]	LA27	LPS	Truncated aptamer LA27 combined with GO-based FP assays
Salmonella [20]	Apt-*E. coli* Apt-S. typ	*E. coli* S. typ	Aptamer modified by fluorescent magnetic multifunctional nanoprobe (APT-FMNPs)
*S. aureus* [21]	Chimeric *S. aureus* aptamer	Staphylococcal enterotoxin	Fluorescent detection based on finely designed chimaera aptamer, MB elements and chain displacement
*L. monocytogenes* [22]	LM6-116	Members of Listeria genus	Two-site binding sandwich assay
*E. coli* [23]	Aptamers for *S. enterica*, *E. coli* and *L. monocytogenes*	*S. enterica*, *E. coli*, *L. monocytogenes*	Multiplexed bar-chart SpinChip integrated with PtNPs-mediated magnetic aptasensor
*E. coli* [24]	E1	*E. coli* O157:H7	Aptamer binding microchip capillary electrophoresis coupled with laser-induced fluorescence (MCE-LIF)

**Table 3.** Targets of pathogenic viruses for development of specific aptamers.

Type of viruses	Aptamer	Target	Method
Human immunodeficiency virus (HIV) [11]	HIV ssDNA aptamer	HIV-1 gene	Molecularly imprinted polymer electrochemi- luminescence sensor
HIV [28]	AntiTat	HIV-TAT protein	Spectroscopic ellipsometer
Hepatitis B virus [30]	A thiol-modified aptamer against HBsAg	HBsAg	Chemiluminescent aptamer-sensor based on rapid magnetic separation
Hepatitis C virus [32]	Core-speciﬁc DNA aptamer	HCV core antigen	Electrochemical aptamer sensor based on graphene quantum dots
Hepatitis C virus [33]	HCVcp ssDNA aptamer	HCVcp	A plasmonic nanoplatform
Human papillomavirus [34]	HPV16 L1 ssRNA aptamer	HPV16 L1 protein	The laser desorption/ionization mass spectrometry method
Human papillomavirus [35]	HPV-07	HPV16 virus-like particle (VLP)	Slow abscission rate-modified DNA aptamer
Influenza [37]	NP-speciﬁc aptamer, CTnI-speciﬁc aptamer	Inﬂuenza nucleoprotein (NP) and cardiac troponin I (cTnI).	Paper-based transverse flow immunoassay (LFIA) using sandwich immunoassay
SARS-CoV [43]	ssDNA	nucleocapsid protein	Aptamer-based Western blot analysis
Norovirus [40,41]	Aptamer-6-FAM, Bt-Apt-Fc	norovirus capsid protein, VP1,	Paper-based microfluidic device or microﬂuidic platform integrated with graphene-gold nano-composite aptasensor
SARS-CoV-2	Not available	Spike protein	To be developed

## Bacterial infection

Aptamers against bacteria are divided into the following two categories (A) targeting predetermined bacterial cell surface antigens or bacterial virulence factors; (B) targeting whole cells with known or unknown molecular targets. So far bacterial aptamers are mainly designed to target Mycobacterium tuberculosis (M. TB), Salmonella, *Listeria monocytogenes* (*L. monocytogenes*), *Staphylococcus aureus* (*S. aureus*), and *Escherichia coli* (*E. coli*) (Table 2).

### Tuberculosis

Tuberculosis (TB) remains one of the leading causes of death worldwide. Traditional diagnostic methods, including tuberculin skin test, tuberculosis antibody detection, and M. TB microscopic examination, have some shortcomings, such as low sensitivity, poor specificity, long-term process, false positive, or negative results. There is an urgent demand to develop reliable, sensitive, stable, and less costive tests for early diagnosis of tuberculosis in resource-deficient communities. To achieve rapid and secure methods for early diagnosis in resource-deficient communities, two diagnostic tests based on DNA aptamers were developed, namely aptamer connection fixation adsorption analysis (AptamerAlisa) and electrochemical sensor (ECS), for tuberculosis biomarker HspX in sputum [9]. A blinded clinical study in 314 sputum samples demonstrated that the sensitivity of AptamerAlisa (94.1%) was better than the anti-HspX polyclonal antibody-based ELISA (68.2%), without affecting the detection specificity of 100%. It was proven that aptamer-based detection was superior to smear microscope, antibody-based ELISA, and chest X-ray in the detection of tuberculosis. In addition, a 30-minute care-point ECS assay was developed to distinguish between tuberculous and non-tuberculous sputum with 92.3% sensitivity and 91.2% specificity in 57 culture-negative and 13 culture-positive sputum samples. Therefore, the biosensor has potential usefulness in detecting active cases in high-risk groups and in screening for tuberculosis in suspected subjects.

Synthetic aptamers combined with co-adsorbents on a gold electrode were optimized to detect secretory protein MPT64, which is a highly immunogenic peptide and pathogenic factor of M. TB, with a detection limit of 81 pMol in serum samples, and reduce the detection time from a few days or hours to 30 min [18]. A new sandwich electrochemical body-fitted sensor was also developed for determination of MPT64 [19]. Under an optimal condition, the proposed aptasensor showed a wide linear range for MPT64 from 1 fg/mL to 1 ng/mL with a detection limit as low as 0.33 fg/mL, which indicates a broad usage in clinical diagnosis of tuberculosis.

Taking together, aptamer-based innovations hold a great potential as rule-in as well as rule-out screening tests with high sensitivity and specificity. The maturation and translation of these innovations would aid in high-throughput and on-site screening of TB infection in communities with disparity.

### Salmonella

Salmonella infection caused by contaminated food has been going on for decades because hundreds of thousands of patients are infected worldwide each year. Therefore, the detection of salmonella in contaminated food is a critical preventive measure in public health. A broad-spectrum ssDNA aptamer containing 80 nucleotides (LA80) capable of binding to lipopolysaccharide (LPS) from four different sources, was truncated to a 27-nt aptamer (LA27) and then developed to detect LPS from *salmonella typhimurium* [10]. The results showed that the aptamer LA27 retained the broad-spectrum ability and had high affinity (K_D_ = 46.2 ± 9.5 nM). Fluorescent polarization analysis was designed with 6-carboxyfluorescein (FAM)-labeled LA27. The sensitivity of FAM-labeled LA27 probe was enhanced dramatically (5–29-fold) with its detection limits of 38.7, 88.0, and 154 ng/mL, respectively.

In order to improve the methods of bacterial isolation and collection, a magnetic and quantitative fluorescent strategy based on aptamer-modified magnetic multifunctional nanoprobe (APT-FMNPs) was developed to simultaneously separate multiple pathogens [20]. The quantitative analysis was carried out within 1 h with a detection limit of 150 cfu/mL for *S. typhimurium* in milk, 120 cfu/mL in human serum and urine. Thus, APT-FMNPs may effectively detect a variety of pathogens in different features of samples.

### Staphylococcus aureus

*S. aureus* is one of the major pathogens in humans and is responsible for a wide range of infection, including hospital-acquired infection and life-threatening newborn infection. Moreover, the bacteria may cause a foodborne illness due to the excretion of several enterotoxins. For a quick detection in biological specimens, ssDNA aptamers against staphylococcal enterotoxin (SES) have been selected for a fluorescence assay in quick detection of *S. aureus* [21]. The chimaera sequence consists of the aptamer sequence against *S. aureus* and the complementary sequence. In order to improve the sensitivity, the signal was amplified by target recovery based on chain displacement. This method exhibited a linear correlation between the fluorescent intensity and the logarithm of *S. aureus* concentration in the wide concentration range from 80 to 8 × 10^6 ^CFU/mL. The lowest detection limit is 39^ ^CFU/mL. The method was applied to detect S. aureus in different water samples with satisfactory recovery and reproducibility.

### Listeria monocytogenes

*L. monocytogenes* is a foodborne Gram-positive bacterial pathogen that causes listeriosis, a lethal disease. The bacterium may survive and grow in a wide range of environmental conditions, including cooling temperatures (4°C). Both the US FDA and the European Medicines Agency have implemented a zero-tolerance policy against *L. monocytogenes* in ready-to-eat food, leading to extensive efforts to develop highly sensitive biosensors for the detection of foodborne pathogens. A double-site binding sandwich method was established to detect the pathogen by specific single-strand DNA aptamer [22]. Antibody-immobilized immunomagnetic beads are used to capture *L. monocytogenes* and then expose them to aptamer probes. The detection was achieved by PCR amplification combined with aptamer. The low detection limit is 5^ ^CFU/mL. Applied to cooked turkey meat, this sandwich method gave rise to positive results when the initial inoculation level was 1–2 log_10_CFU per 25^ ^g. Due to a very low detection limit, the method is applicable in detecting *L. monocytogenes* in food and environmental samples.

### Escherichia coli

*E. coli* is a widely distributed pathogen, therefore it is necessary to establish a rapid, simple and accurate detection method. A portable multiplexing bar graph rotation chip (MB-SpinChip), which integrates nanoparticle-mediated magnetic sensors, was developed for the quantitative detection of a variety of pathogens [23]. This multifunctional multiplexed SpinChip combines aptamer-specific recognition with nanoparticle-catalysed pressure amplification to achieve sample-to-answer output for sensitive point-of-care testing (POCT). This is the first report to use a volume bar chart chip for pathogen detection, and it is also the first to use a “rotation” mechanism for multiplex bar chart detection. This user-friendly MB-SpinChip allows simultaneous and quantitative detection of three main foodborne pathogens including *S. enteritidis*, *E. coli*, and *L. monocytogenes* in apple juice with a detection limit of about 10 CFU/mL, without the need for any specialized instruments. This MB-SpinChip has the visually quantitative reading based on bar chart, holds the great potential to simultaneously detect all kinds of pathogens, and bears a wide range of applications in food safety, environmental monitoring and screening of infectious agents.

A specific method was developed for the detection of E. coli using bacterial-specific aptamer binding microchip capillary electrophoresis coupled with laser-induced fluorescence (MCE-LIF) [24]. Under an optimized condition, the detection limit was 3.7 × 10^2 ^CFU/ml. The recovery rates in drinking water and milk samples were 94.7% and 92.8%, indicating that this strategy is useful for the detection of E. coli in food and the environment. As surface-enhanced Raman scattering (SERS) signal is significantly enhanced through its special recognition aptamer, bacteria are directly and visually identified by SERS spectrum with an improved detection limit (1.5 CFU/mL) [25].

## Viral infection

Many diseases are caused by viral infection, such as influenza, hepatitis, acquired immunodeficient syndrome (AIDS), or emerging viral diseases, such as Ebola, viral pneumonia caused by MERS, SARS-CoV or SARS-CoV-2 infection, which lead to severe illness and death, as well as tremendous economic and social impacts. Rapid and safe diagnosis of viral infection is one of the critical factors in controlling broad transmission and treating infected individuals [26]. Viral proteins involved in adherence, penetration, replication, and release in the replicative cycle are specific targets for aptamer selection and optimization. At present, a number of aptamers have been reported to hold potential applications in the diagnosis of viral infections, such as human immunodeficiency virus-1 (HIV-1), hepatitis B virus (HBV), hepatitis C virus (HCV), human papillomavirus (HPV), influenza virus, norovirus, or SARS-CoV (Table 3). A fast and sensitive detection kit with high specificity is urgently desirable for global SARS-CoV-2 infection in a large population.

### Human immunodeficiency virus

HIV is the main cause of AIDS, and leads to significant morbidity and mortality. As highly effective antiretroviral therapy (HAART) cannot completely eradicate silent viruses, much attention has been paid to the early detection of HIV and the pathophysiologic mechanisms of controlling reactivation during or after HAART therapy. Several crucial viral proteins such as Gp120, Gag polyprotein, Rev and enzymes like reverse transcriptase (RT), integrase, and proteases have been considered to be the targets of detection [27]. A novel molecularly imprinted polymer (MIP) electrochemiluminescence (MIP-ECL) sensor was developed for the detection of highly sensitive and selective HIV-1 genes using europium sulphide nanocrystalline (ESNC) as signal generation compounds [11]. With MIP-ECL and ESNC strong electronic chemiluminescent emission, the sensitive and selective detection of HIV gene was achieved in the linear range of 3.0 fMol∼0.3 nMol, and the detection limit was 0.3 fMol. Compared to non-complementary sequences and two base mismatch sequences, the current MIP-ECL biosensors demonstrate high specificity in HIV gene detection. The proposed ECL biosensor has been applied to the detection of HIV proviral DNA in human serum samples with satisfactory results. Because of its high sensitivity, selectivity, repeatability, and stability, ESNCs may be used as a new luminescent probe to develop MIP-ECL sensors for the detection of other DNA biomarkers.

HIV-TAT (trans transcriptional activator) protein-specific RNA aptamer (AntiTat) and spectral ellipsometer are preferred to improve the specificity and sensitivity of diagnosis [28]. It is reported for the first time that anti-Tat aptamers were able to detect the protein by both spectroscopic ellipsometry (SE) and surface plasmon resonance-enhanced total internal reflection ellipsometry (SPReTIRE). The detection limit of anti-Tat aptamers was within a range of nMol-pMol of the protein in buffer solution.

### Hepatitis B and C viruses

Hepatitis B virus (HBV) is a partial double-stranded DNA virus of the Hepadnaviridae family, which is divided into eight genotypes from A to H. The most characteristic component of HBV viral protein is hepatitis B surface antigen (HBsAg) [29]. HBsAg-specific aptamer was selected to develop a novel chemiluminescent aptamer-sensor based on rapid magnetic separation and bifunctional gold nanoparticles [30]. The sensor recognizes rapid magnetic separation and attains high sensitivity for the detection of HBsAg in HBV-positive serum. The detection limit is 0.05 ng/mL, which is much lower than 0.5 ng/mL of the typical ELISA used in clinical services.

The hepatitis C virus (HCV) is a common cause of chronic hepatitis, cirrhosis, and hepatocellular carcinoma (HCC), and there is no approved vaccine. Ten viral proteins were produced from a 3000-amino-acid polyprotein during the maturation mediated by protease, and these structural or nonstructural proteins or viral enzymes are the targets of specific aptamers [31]. Aptamer-based biosensors allow the detection of HCV infection at an early stage. By an aptamer proximity binding strategy, a novel electrochemical biosensor was designed for supersensitive detection of HCV core antigen [32]. The immobilized surface was prepared by modifying glassy carbon electrode (GCE) with graphene quantum dots (GQD). Through π-π stacking, the richness of hydrophilic edge and hydrophobic surface in GQD and the hydrophobic plane in GQD enhance the adsorption of aptamer on the electrode surface, thus introducing a new substrate suitable for nucleic acid aptamer. Electrochemical impedance spectroscopy (EIS), cyclic voltammetry (CV), and differential pulse voltammetry (DPV) were performed at each stage of the chemical modification to confirm the resulting surface changes. With EIS as an effective alternative system for the detection of the core antigen, two linear concentration ranges are 10–70 and 70–400 pg/ml with a detection limit of 3.3 pg/mL. In addition, the sensor accurately detects HCV core antigen in human serum samples. This nucleic acid sensor represents a rapid, selective, and sensitive strategy for the detection of HCV core antigen in clinical diagnosis.

In a recent study, a plasma nanoplatform was constructed by using hepatitis C virus core protein (HCVcp), G-quadruplex/hemin DNAzyme and nanofibrous membrane as hairpin to assemble amplification reaction [33]. HCVcp was detected in whole serum with naked eye at an ultra-low concentration of 1.0 × 10^−4 ^pg/mL. By testing serum samples from 30 donors with different viral loads, the plasma nanoplatform has demonstrated high specificity, excellent reusability, long-term stability and better detection sensitivity than a commercial ELISA kit. Naked eye detection based on plasma nanoplatform is expected to have potential application in the early diagnosis of hepatitis C and other infectious diseases.

### Human papillomavirus

Human papillomavirus (HPV) is a DNA virus of the papillomavirus family. Most HPV infection does not cause symptoms and subside themselves. However, some of them cause persistent infection and warts or precancerous lesions, increasing the risk of cancer in the cervix, vulva, vagina, penis, anus, mouth or throat [26].

The detection of HPV-L1 protein provides information about the status of viral infection, reflects the viral replication in cervical cells, and determines the regression and progression of cervical lesions. A new laser desorption/ionization mass spectrometry (LDI MS) method was reported for sensitive detection of HPV-16-L1 protein based on non-covalent competition between HPV-16-L1 aptamers and melamine adsorbed on gold nanoparticles (AuNPs) [34]. The intensity of the MS signal corresponding to the mass tag has a linear relationship with the HPV-16-L1 concentration within 2–80 ng/mL range, and the detection limit is 58.8 pg/mL. Using this method, the HPV-16-L1 protein in clinical and vaccine samples was quantitatively analysed. The method exhibits the advantages of simplicity, high sensitivity, and good reliability.

Several problems of antibodies like batch variability, limited stability, and long discovery lead time were overcome by developing slow abscission rate-modified DNA aptamer (SOMAmer), which targets antigens in the HPV vaccine [35]. An HPV-07 aptamer was selected to bind to the type 16 virus-like particle (VLP) formed by the self-assembled capsid protein L1. The HPV-07 aptamer binds to a similar or closely related epitope as the neutralizing antibody H16.V5, indicating at least part of the recognition of clinically relevant epitopes. When used in an ELISA format, the aptamer exhibited both high precision (with a medium precision of 6.3%) and a large linear range from 25% to 200% of the typical formula. In order to make further use of the aptamer, a simplified hybrid assay is developed. Compared with the traditional ELISA, this assay provides a significant reduction in time. These results demonstrate that aptamers are suitable reagents for biotiter determination, and hopefully their implementation improves the current determination effectively.

### Influenza

Influenza is considered to be the most prevalent infectious disease in human beings. New influenza viruses caused major three pandemics in twentieth century [26]. In fact, it is estimated that the Spanish flu virus killed 25 million people [36] around the world. The rapid detection and classification of influenza viruses are particularly important because these viruses have a high risk of infection and high-frequency mutations, which often lead to the arrival of new strains, resulting in epidemics and even pandemics. Paper-based lateral ﬂow immunoassays (LFIA) using traditional sandwich immunoassay is one of the most commonly used hospice tests. The application of gold nanoparticles (AuNPs) in LFIA cannot meet the sensitivity requirements for the detection of infectious agents or biomarkers at low concentration in body fluids because of the number of AuNPs that bind to the target is limited. In order to overcome this problem, a single-stranded DNA-binding protein (DNA-binding domain of RPA70A, human replicative protein A70kDa A) was acquired, which uses capture antibodies and aptamers fixed in LFIA as probes for sandwich detection [37]. Thus, the signal strength was enhanced by connecting several AuNPs to each aptamer. This method was to detect influenza nucleoprotein (NP) and cardiac troponin I (CTnI). The detection limits of AuNP in human nasal fluid and CTnI in serum were 0.26 and 0.23 pg/mL, respectively. This technique demonstrates significantly higher sensitivity than conventional methods that are widely used in LFIA involving antibody binding to AuNPs. These results underscore that the proposed method is widely applicable in the detection of highly sensitive substances in the field of POC for early diagnosis.

### Norovirus for acute gastroenteritis

Human norovirus (NoV) is the leading cause of acute viral gastroenteritis worldwide, and often causes foodborne illness resulting in large and persisting outbreaks. It is pathogenic and highly contagious; however, no vaccine is currently available. The noroviral genome is positive single-stranded RNA enclosed in an icosahedral capsid consisting of capsid viral protein 1 (VP1) and VP2. The capsid is thermal-resistant, allowing the virus to survive in temperatures up to 55°C and a pH range of 3–7. The current diagnostic method is nucleotide-based RT–PCR detection of the viral RNA. However, one kit may not cover different genetic strains of the virus, and it is less convenient for point-of-care detection. To meet this need, specific aptamer against VP1 was identified [38], and the SELEX screening stringency was enhanced by adding food matrices in the system in order to enhance the aptamer specificity against viral P domain containing the outermost domain of the VP1 capsid protein [39]. A highly sensitive point-of-care device for rapid determination of noroviruses was successfully developed with a previously characterized aptamer against NoV [40]. An easy-to-make paper-based microfluidic platform was further developed using a nitrocellulose membrane and integration of 6-FAM-labeled aptamer. The noroviral quantitation was successfully performed with a linear range from 13 ng/mL to 13 μg/mL of norovirus and detection limits at 3.3 ng/mL. The device is simple and cost-effective, and holds great potential of rapid *in situ* visual determination of noroviruses with desirable sensitivity and specificity. Hence, this aptasensor- and paper-based microfluidic device provides a new method for early identification of norovirus. Moreover, a microﬂuidic platform integrated with graphene-gold nano-composite aptasensor was reported [41], and the latter had a detection limit of 100 pM with a detection range from 100 pM to 3.5 nM for norovirus. Therefore, the aptamer-based noroviral detection is evolving, and it is anticipated that a point-of-care detection, such as lateral flow assay [16] may be developed for a broad detection of this or other contagious viruses.

### SARS-CoV and SARS-CoV-2 viruses

The pandemics of SARS-CoV in 2004 and current SARS-CoV-2 in more than 50 countries resulted in global infection in more than two million cases, 137,000 death so far (by April 16, 2020). Most detection kits used in establishing the infection are nucleotide-based RT–PCR assays, however, a high false-negative rate (approximately 40%) has hampered the quick estimation of infected individual number and clinical diagnosis. Therefore, quick, simple, reliable detection methods are in urgent need to timely report actual infected subjects, monitor efficiency of preventive measures, and determine the efficacy of therapeutic strategies and eradication of viral presence. ssDNA-aptamer was selected for detection of SARS-CoV nucleocapsid N protein with a K_D_ value of 4.93 ± 0.30 nM [42]. The aptamer-based Western blot analysis was verified by regular ELISA to be a rapid and sensitive method in the detection of SARS-CoV protein for infection validation. With an illustration of spike protein 3D crystal structure [43] and being familiar with the aptamer-based biosensor technologies [17], aptamer against the receptor-binding domain of the SARS-CoV-2 spike glycoprotein has been already identified [8], and an aptamer-based novel detection could be developed for the investigation of ongoing COVID-19 pandemics and clinical service.

## Protozoan parasites infection

Infectious diseases caused by protozoa parasites affect hundreds of millions of people in underdeveloped and developing countries, and remain a major health problem worldwide. These parasitic infections are usually transmitted through insect vectors or contaminated food and water, and their high prevalence is mainly related to poor sanitary conditions or an immunodeficient status. At present, the primary diagnostic methods include the growth and microscopic observation of pathogens in clinical specimens, the immune detection of parasite proteins with antibodies, or the identification of pathogen genomes by PCR. However, these technologies have several limitations because they are time-consuming, and require cryogenic storage conditions and specialized instruments, which are hardly available in remote areas where most infected people live. Therefore, a great deal of research has focused on the identification of new targets from the parasites. In this case, aptamer has become an attractive tool for developing alternative diagnostic tools (Table 4).

**Table 4.** Targets of pathogenic parasites for development of specific aptamers.

Type of parasites	Aptamer	Target	Method
Trypanosoma cruzi [11]	Apt68	Mammalian stage trypomastigotes	Streptavidin paramagnetic beads coated with biotinylated Apt68
Leishmania [28]	LmWC-25R; LmHSP 7b/11R	Leishmania promastigote protein extracts	Fluorescent enzyme-linked DNA aptamer-magnetic bead sandwich assay
Plasmodium for malaria [30]	P38 or NG3	Plasmodium lactate dehydrogenase and plasmodium falciparum glutamate dehydrogenase	Dye coupled aptamer-captured enzyme catalysed reaction

### Trypanosoma evansi

*T. evansi* is a blood-borne parasite and the pathogen of Chagas disease, and its genome is detectable in the blood of infected individuals using PCR-based methods. However, shortly after a natural infection or during the chronic phase of the infection, number of the parasites in the blood may be so low that it is difficult to detect with PCR. With a whole cell SELEX strategy, serum-stable RNA aptamers were developed to bind to live T. cruzi trypomastigotes [44]. Apt68-coated paramagnetic beads were used to pull down and concentrate parasites from samples with low levels of parasites, and facilitate subsequent detection by PCR. These aptamers bound to parasites with a strong affinity (8-25 nM range), and exhibited a high specificity to the target that is only expressed in on *T. cruzi* trypomastigotes. The specificity is endorsed by the fact that the aptamer did not interact with the epistatic parasites of epimastigotes of *T. cruzi*, nor with other related trypanosomal parasites, Donovanni and Trypanosoma brucei. Biotinylated Apt68 was fixed on the solid phase and captured living parasites. These captured parasites are visible under the microscope and form as large active aggregates when aptamers are coated with paramagnetic beads to bind to the surface of Trypanosoma. In addition, Apt68 was able to capture and aggregate trypanosomes from several isolates of the two main parasite genotypes. With magnet force, these parasite-bead aggregates are separated from whole blood samples with parasite spines, even if the concentration is as low as five parasites in 15 millilitres of whole blood. These results demonstrated that aptamers could be used as pathogen-specific ligands to capture and facilitate the detection of *T. cruzi* in blood.

### Leishmania

A fluorescent peroxidase linked DNA aptamer-magnetic bead sandwich method was invented to detect soluble proteins extracted from the main proflagellates of Leishmania [45]. The protein was extracted with high molar concentration of chiral salt. This study employed nanoparticles and quantum dots to bind Amplex Ultra Red for fluorescent detection. Although all versions are highly sensitive, AUR-based versions show low variability between samples. This highly portable and rechargeable battery-operated fluorometer with an on-board computer allows a rapid (<1 h) and sensitive detection of leishmania proflagellate protein extract (100 ng/sample) in sand fly homogenate in remote areas, and is capable of mapping the geographical distribution of the main parasites in wild sand fly population.

### Plasmodium for malaria

There is a great demand for accurate diagnosis of malaria. The detection of pan-malaria and plasmodium falciparum was achieved by dye reaction catalysed by plasmodium lactate dehydrogenase (PLDH) and plasmodium falciparum glutamate dehydrogenase (PFGDH), respectively [46]. The aptamer-captured parasite PLDH and PFGDH enzymes were detected by substrate-dependent reaction combined with the conversion of Resazurin (blue, *λ* 605 nm) to Resorufin (pink, *λ* 570 nm) dye. The reaction was monitored by measuring fluorescent intensity, absorbance ratio (*λ* 570/605 nm) and colour change (blue to pink) of resorcinol at *λ* 660 nm. The detection method is customized to different spectrophotometers. For these two methods, biomarkers are captured from serum samples with aptamer-coated magnetic beads before analysis to eliminate potential interference from serum. In the instrument-less device, a medical syringe (5 mL) prefabricated with a magnet is used for *in situ* separation of enzyme-captured beads from the reaction supernatant. Then, the converted dye in the supernatant is effectively adsorbed on the diethyl amino ethyl (DEAE) cellulose-treated paper core assembled in the syringe hose. The plasmodium biomarkers are detected in qualitative and quantitative formats according to the colour and pixel intensity developed on the paper surface, respectively. The novel techniques provide the detection of specific biomarkers in a clinically relevant dynamic range, and the detection limit is at a micromolar level. Flexible capability, low cost, non-interference, and portable (for non-instrumental equipment) are the main advantages of this detection.

## Perspectives and conclusions

As an emerging molecular probe, aptamer-based technology has been used as an alternative to antibodies in developing aptamer-based biosensors (aptasensors) for detection of microbial components in a specific, quick, and sensitive fashion, and some of them are operative in remote areas in meeting the need for point-of-care testing. Through a SELEX screening and enriching process, target-specific aptamers are selected with high efficiency, and are flexible for conjugation of a variety of sensor molecules to facilitate chemiluminescent or fluorescent measurement, to link them to solid phases or nanoparticles for visualization or capture. Multifaceted options for modification endow aptasensors with diversely available conﬁgurations, including electrochemical, fluorescent, chemiluminescent, or mass-sensitive, etc. Moreover, for emerging viral pathogens, such as, influenza, SARS-CoV, and SARS-CoV-2 in a pandemic period, a quick, simple, and reliable approach is in an urgent need for mass population screening of infected individuals. Aptasensor-based technologies for detecting biomarkers at pMol levels enable such a screening or diagnosis possible at a low cost in point-of-care sites. The expansion of aptasensor-based technologies in the infectiology field to other areas, such as food hygiene and environmental monitoring would provide a feasible means of distal monitoring at various sites in a simultaneous and timely mode.

Application of aptamer-based detection has not been as widely as antibodies in the biomedical field. The main reason for this probably lies with the complexity to select highly-specific aptamers for a particular target, such as bacterial or viral components on an individual base. In most cases, microbial pathogens, their enzymes, structural or nonstructural proteins are the targets for the development of specific aptamers. However, when bacterial or viral targets bear significant negative charges, it will enhance the difficulty to enrich highly-specific aptamer candidates through an SELEX process [47], because oligonucleotide-based aptamers have negative charges on their backbone [48]. To obtain highly-specific aptamer candidates, the stringency of a SELEX selection process is critical, and negative and countermeasures may be often introduced to exclude low affinity or non-specific aptamers [49]. At the same time, an introduction of additional counter steps might have a negative impact by increasing the time and cost for an overall selection process [7]. Furthermore, enriched aptamers need a rigorous validation and optimization for target specificity and sensitivity. The additional obstacles for aptamer-based biosensors are the stability of aptamers, because nucleotide aptamers will be subjected to degradation by ribonuclease in the serum, although special modification, for instance, phosphorothioate modification, may enhance the resistance to nuclease degradation. Currently, most immunological or microbiologic laboratories are equipped with instruments that are specially designed for antibody-based detection. For switching to aptamer-based detection requires pharmaceutical industries, clinical, and research laboratories for new investments on both technology development and equipment design and validation. Such a transition is happening, although slowly. In spite of all challenges mentioned above, compelling advantages over antibody-based assays, such as relatively low cost for manufacture, multiplexity, and high throughput in nature, have pushed the maturation of aptamer-based technologies and translation to clinical practice, especially in mass screening for pandemic transmission of emerging pathogens, such as SARS-Cov-2 in heavily affected areas. It is anticipated that both antibody- and aptamer-based technologies will be widely utilized in the detection of microbial components for research, epidemical investigation and clinical services or food and environmental monitoring with almost equal specificity and sensitivity. Ultimately, aptamer-based technologies may be more acceptable for point-of-care testing due to their more flexibility for modification and stability in a warm or hot atmosphere.

In conclusion, taking the advantages of high sensitivity, reproducibility, specificity, and time-saving, novel aptamer-based technologies are considered as the most promising methods to detect a wide range of pathogens and biochemical components in food safety, environment surveillance, and diagnosis of infectious diseases at the point-of-care and resource-poor settings. As emerging technologies, their maturation and translation to the clinical application need rigorous optimization and validation for the high quality of service demanded in clinical practice.

## Abbreviations

**Abbreviations**: AIDS: acquired immunodeficiency syndrome; AST: antimicrobial sensitivity test; APT-FMNPs: aptamer-modified fluorescent magnetic multifunctional nanoprobe; AuNPs: gold nanoparticles; CTnI: cardiac troponin I; CV: cyclic voltammetry; DEAE: diethyl amino ethyl; DPV: differential pulse voltammetry; EIS: Electrochemical impedance spectroscopy; ECS: electrochemical sensor; *E. coli*: *Escherichia coli*; ESNC: europium sulphide nanocrystalline; GCE: glassy carbon electrode; GQD: graphene quantum dots; HBsAg: hepatitis B surface antigen; HBV: hepatitis B virus; HCV: hepatitis C virus; HCVcp: hepatitis C virus core protein; HCC: hepatocellular carcinoma; HAART: highly active antiretroviral therapy; HIV-TAT: human immunodeficiency virus trans transcriptional activator; HIV-1: human immunodeficiency virus-1; HPV: human papillomavirus; LDI MS: laser desorption/ionization mass spectrometry; LFIA: lateral ﬂow immunoassays; LOD: limit of detection; LPS: lipopolysaccharide; MCE-LIF: microchip capillary electrophoresis coupled with laser-induced fluorescence; MB: molecular beacon; MIP-ECL: molecularly imprinted polymer electrochemiluminescence; MB-SpinChip: multiplexing bar graph rotation chip; M. TB: Mycobacterium tuberculosis; NC: nucleocapsid; NP: nucleoprotein; PFGDH: plasmodium falciparum glutamate dehydrogenase; PLDH: plasmodium lactate dehydrogenase; POCT: point-of-care testing; RT–PCR: reverse transcriptase polymerase chain reaction; SOMAmer: slow abscission rate-modified DNA aptamer; SE: spectroscopic ellipsometry; SES: staphylococcal enterotoxin; *S. aureus*: *Staphylococcus aureus*; SPReTIRE: surface plasmon resonance-enhanced total internal reflection ellipsometry; SERS: surface-enhanced Raman scattering; VLP: virus-like particle.
